# SARS-CoV-2 Virus-like Particles (VLPs) Specifically Detect Humoral Immune Reactions in an ELISA-Based Platform

**DOI:** 10.3390/antib11040076

**Published:** 2022-12-12

**Authors:** Stefan Hirschberg, Hannes Bauer, Julian Kamhieh-Milz, Frauke Ringel, Christoph Harms, Omar Kamal Eddin, Axel Pruß, Katja Hanack, Kai Schulze-Forster

**Affiliations:** 1 Charité-Universitätsmedizin Berlin, Corporate Member of Freie Universität Berlin, Humboldt-Universität zu Berlin, and Berlin Institute of Health, Institute of Transfusion Medicine, 10117 Berlin, Germany; 2 CellTrend GmbH, 14943 Luckenwalde, Germany; 3 Wimedko GmbH, 12101 Berlin, Germany; 4 DHS—Diagnostic HealthCare Solutions GmbH, 13347 Berlin, Germany; 5 Charité-Universitätsmedizin Berlin, Corporate Member of Freie Universität Berlin, Humboldt-Universität zu Berlin, and Berlin Institute of Health, Center for Stroke Research Berlin, Department of Experimental Neurology, 10117 Berlin, Germany; 6 Department of Biochemistry and Biology, University of Potsdam, 14476 Potsdam, Germany

**Keywords:** virus-like particle (VLP), SARS-CoV-2, in vitro diagnostic device (IVD), enzyme-linked immunosorbent assay (ELISA), immune reaction, antibodies

## Abstract

A key in controlling the SARS-CoV-2 pandemic is the assessment of the immune status of the population. We explored the utility of SARS-CoV-2 virus-like particles (VLPs) as antigens to detect specific humoral immune reactions in an enzyme-linked immunosorbent assay (ELISA). For this purpose, SARS-CoV-2 VLPs were produced from an engineered cell line and characterized by Western blot, ELISA, and nanoparticle tracking analysis. Subsequently, we collected 42 serum samples from before the pandemic (2014), 89 samples from healthy subjects, and 38 samples from vaccinated subjects. Seventeen samples were collected less than three weeks after infection, and forty-four samples more than three weeks after infection. All serum samples were characterized for their reactivity with VLPs and the SARS-CoV-2 N- and S-protein. Finally, we compared the performance of the VLP-based ELISA with a certified in vitro diagnostic device (IVD). In the applied set of samples, we determined a sensitivity of 95.5% and a specificity of 100% for the certified IVD. There were seven samples with an uncertain outcome. Our VLP-ELISA demonstrated a superior performance, with a sensitivity of 97.5%, a specificity of 100%, and only three uncertain outcomes. This result warrants further research to develop a certified IVD based on SARS-CoV-2 VLPs as an antigen.

## 1. Introduction

The first cases of a new pulmonary disease were reported from the province of Wuhan in China in 2019. The severe acute respiratory syndrome coronavirus 2 (SARS-CoV-2) caused the disease COVID-19 and the ongoing pandemic [1,2]. While the majority of infected people develop only mild flu-like symptoms or are asymptomatic, the disease COVID-19 poses a serious risk of hospitalization or death to susceptible individuals [3,4]. So far, the most widely used measures to contain the spread of the virus have focused on the direct detection of the virus via polymerase chain reaction (PCR) or antigen-based methods and a strict quarantine regime. With a global decrease in the number of severe cases and a reduction in the number of tests used to screen contagious individuals, the serological determination of immune status will become more important in determining the risk for an individual or a population.

SARS-CoV-2 consists of four major structural proteins. The spike glycoprotein (S) mediates the internalization and contains a high-affinity receptor-binding domain (RBD) for the human angiotensin-converting enzyme 2. The nucleoprotein (N) is involved in the packaging of the genome. The viral vesicular structure is formed by the membrane (M) and the envelope (E) proteins [5,6]. The simultaneous expression of SARS-CoV-2 structural proteins leads to the spontaneous assembly of virus-like particles (VLPs) [7,8]. VLPs are multi-protein structures that resemble certain viruses in their molecular composition and shape. Free of genetic material, these particles are not infectious and are therefore safe to handle.

In the US, 77 serology-based tests have an active Emergency Use Authorization from the Food and Drug Administration (last updated 30 March 2022). The vast majority of these tests only measure antibodies against the spike protein or the RBD. Only 13 tests contain peptides or the full-length recombinant nucleoprotein. It has been proposed that presenting peptides from more than one protein increases the sensitivity of serological tests [9]. However, there are only nine FDA-authorized tests that contain peptides from two proteins (N and S) and only one test that contains any peptides of the M-, N-, and S-protein. Although quite common for other viral diseases [10,11,12,13,14], to our knowledge, there is no serological test that utilizes VLPs as antigens to detect antibodies against SARS-CoV-2.

During this pandemic, SARS-CoV-2 VLPs were mostly developed as vaccine candidates [15,16,17,18] or as research tools to monitor viral entry into susceptible cells [19,20,21,22]. Surprisingly, SARS-CoV-2 VLPs have attracted very little attention in the diagnostic field.

In this proof-of-principle study, we characterized the reactivity of SARS-CoV-2 VLPs containing all four major proteins with serum from a cohort of 230 individuals. We found that high sensitivity and specificity as well as the superior performance of the VLP-based ELISA in comparison to a certified IVD validate the utility of SARS-CoV-2 VLPs as antigens to detect specific immune reactions in human serum.

## 2. Materials and Methods

*Serum collection and ethics statement:* Serum samples were obtained from different sources, such as patients of a private in-and out-patient clinic (MeoClinic, Berlin), the outpatient clinic of a physician (Dr. Omar Kamal-Eddin), from lab members and relatives, as well as in part due to the service of the Diagnostic HealthCare Solutions GmbH. Ethics approval was obtained by the ethical review board of the Charité—Universitätsmedizin Berlin (EA1/304/21) and all participants provided written consent to participate in this study. Blood was processed on the same day of withdrawal by centrifugation at 4500× *g* for 10 min at 4 °C. Serum samples were stored at −20 °C for short-term storage and at −80 °C for long-term storage. 

*Culturing of Expi293 cells:* The Expi293™ Expression System Kit, composed of the Expi293 suspension adapted cell line, Expi293 transfection reagents, and Expi293 culture medium, was purchased from Thermo Fisher Scientific. The cultivation and transfection of Expi293 cells were mostly performed according to the manufacturer’s instructions. Cells were seeded at a density of between 0.3 × 10^6^ and 0.5 × 10^6^ cells/mL and subcultured at a concentration of 3 × 10^6^ to 5 × 10^6^ viable cells/mL after 3–5 days. Cell diameter, the percentage of viable cells (vitality), and the concentration of viable cells were routinely measured using the LUNA Cell Counter (Logos Biosystems, Anyang, South Korea).

*Generation of SARS-CoV-2 VLPs:* Virus-like particles were produced from two genetically modified Expi293 suspension-adapted cell lines. Cells were grown at 37 °C, 8% CO_2_ with 130–150 rpm on a Rotamax120 platform shaker (Heidolph Instruments GmbH & Co. KG, Schwabach, Germany) in Expi293 medium containing a final concentration of 100 units/mL penicillin and 100 µg/mL streptomycin. Cell line 1 (Expi_MEN), contains the human codon optimized M-(Gen Bank: QHD43419.1), E-(Gen Bank: QHD43418.1) and N-genes (Gen Bank: QHD43423.2) stably integrated into the genome. Cell line 2 (Expi_SMEN), additionally contains the S-gene (Gen Bank: QHD43416.1), including the D614G mutation and the R683A and R685A substitution, to render the furin cleavage site (FKO) non-functional, stably integrated into the genome. The induction of VLP production is controlled by the tetracycline-responsive element promoter (TRE) [23] and can be activated by tetracycline or its analogs (e.g., doxycycline) [24,25]. VLP production was induced by the addition of doxycycline-hyclate (Sigma-Aldrich, Taufkirchen, Germany) at a concentration of 1 µg/mL. Approximately 96–120 h after induction, when the vitality of the cell culture was typically 40–60%, supernatants were harvested. Subsequently, cell culture supernatants were cleared by centrifugation at 2000× *g* for 10 min, followed by filtration with a 1.2 µm Minisart NML (Sartorius Stedim Biotech GmbH, Göttingen, Germany) followed by a 0.45 µm Millex Low Binding Durapore (PVDF) syringe filter (Merk Millipore Ltd., Tullagreen, Ireland). Clarified cell culture supernatants were continuously diafiltrated with four times the initial volume of PBS (pH 7.2) at a constant pressure of 0.124–0.165 kPa and 135 rpm using a Minimat EVO Tangential Flow Filtration System equipped with an Omega membrane with a 300 kDa cut-off (Pall Corporation, Dreieich, Germany). VLPs were precipitated from the diafiltrated retentate by the addition of PEG-it Virus Precipitation Solution (System Biosciences, Palo Alto, CA, USA) at a ratio of 1:10. The supernatants were incubated at 4 °C on a rotating shaker for 24–48 h prior to precipitation at 1500× *g* for 30 min. The supernatant was removed, and the VLP-containing pellet was resuspended with sterile PBS (pH 7.2) and stored at −80 °C until use.

*Electron microscopy:* VLP-producing cells, were fixed with 2.5% glutaraldehyde (Serva, Heidelberg, Germany) in 0.1 M sodium cacodylate buffer (Serva, Heidelberg, Germany) for 30 min and postfixed with 1% osmium tetroxide (Science Services, München, Germany) and 0.8% potassium ferrocyanide II (Merck, Darmstadt, Germany) in 0.1 M cacodylate buffer for 1.5 h. Agarose-embedded samples were dehydrated in a graded ethanol series and embedded in Epon resin (Serva, Heidelberg Germany). Finally, ultrathin sections of the samples (70 nm) were stained with 4% uranyl acetate and Reynolds lead citrate [26] (Merck). A Zeiss EM 906 electron microscope at an 80 kV acceleration voltage (Carl Zeiss, Oberkochen, Germany) equipped with a slow scan 2K CCD camera (TRS, Moorenweis, Germany) was used for image acquisition. 

*Nanoparticle tracking analysis (NTA):* NTA was applied to measure the size and concentration of VLPs in different preparations according to our previous protocol [27]. NTA measurements were performed using a NanoSight LM10 instrument (NanoSight, Amesbury, UK) consisting of a conventional optical microscope, a high sensitivity sCMOS camera and an LM10 unit equipped with a 488 nm laser module. The samples were injected into the LM unit via the nanosight syringe pump at a constant flow rate of 50 µL/min using a 1 mL sterile syringe. Sample dilutions of 1:2000 to 1:5000 usually result in an effective particle concentration suitable for analysis with NTA (1.0 × 10^8^ to 2.5 × 10^9^ particles/mL). The capturing settings (shutter and gain) and analyzing settings were manually adjusted and kept constant between all samples that were recorded on the same day. NTA software (NTA 3.2 Dev Build 3.2.16) was used to capture three videos of 30 s each to analyze nanoparticle tracking data for each sample.

*ELISA (Enzyme-linked Immunosorbent Assay):* The protocol was comparable to our previous study [27]. S-protein (Sino Biological Inc., Beijing, China, # 40689-V08B), N-protein (charge 2020/20.7/2, 0.4 mg/mL stock, new/era/mabs GmbH, Potsdam, Germany) or SARS-CoV-2 VLPs were immobilized as antigens on Nunc Polysorb 96-well microtiter plates in 100 µL carbonate buffer per well overnight at 4 °C. Free binding sites were blocked with 250 µL per well of PBS containing 1% bovine serum albumin (BSA) for 45 min at room temperature (RT). After washing with PBS, rabbit monoclonal antibodies against spike- (1:2000, 40689-V08B, Sino Biological Inc., Beijing, China) and nucleoprotein (1:5000, 40143-R019, Sino Biological Inc., Beijing, China) or human serum (1:50–1:100) were incubated with the immobilized antigen in 100 µL PBS containing 0.05% Tween20 (PBS-T) and 1% BSA at RT for 1 h. A detergent-containing buffer was chosen to facilitate access of antibodies to epitopes inside the lumen of the VLPs. After washing, plates were incubated at RT with a horseradish peroxidase (HRP)-conjugated goat anti-rabbit or goat anti-human IgG antibody (Dianova GmbH, Hamburg, Germany) diluted 1:10,000 in PBS-T containing 1% BSA. Unbound conjugated molecules were removed by washing with PBS. The colorimetric reaction using 100 µL tetramethylbenzidine (TMB, Carl Roth GmbH, Karlsruhe, Germany) as a substrate was stopped with 100 µL of 0.125 M H_2_SO_4_ per well after 10–30 min. Absorbance was immediately measured with an Epoch microplate reader at λ = 450 nm and subtracted by the absorbance of the reference wavelength at λ = 620 nm. For the quantitative approximation of N- and S-protein concentrations in VLP samples, the linear range of a 2-log dilution series (triplicate) of the respective proteins was taken as a reference.

*Western blot:* Samples were prepared according to the NuPAGE Technical Guide of Invitrogen. After denaturation in NuPAGE LDS sample buffer with DTT 50 mM for 10 min at 70 °C, the samples and markers were run on a Novex bis-tris gradient gel (4–12%, Thermo Fischer Scientific) using NuPAGE MOPS SDS running buffer and subsequently blotted on a Novex 0.45 µm nitrocellulose membrane (LC2001, Thermo Fischer Scientific). The membranes were blocked for 1 h in PBS containing 1% BSA, and subsequently incubated with a rabbit-anti-nucleoprotein primary antibody (1:5000, 40143-R019, Sino Biological Inc, Beijing, China) or human serum in PBS containing 1% BSA and 0.2% PBS-T at 4 °C overnight on a shaker. Human serum from a double-vaccinated individual (AstraZeneca) was used to detect the S-protein alone or S-protein and N-protein, respectively. High levels of anti-S IgGs were confirmed in the respective human sera by ELISA. HRP-coupled goat anti-human or goat anti-rabbit (Dianova GmbH, Hamburg, Germany) secondary antibodies were incubated at a dilution of 1:10,000 in PBS-T for 3 h at RT. Proteins were detected by chemiluminescence using respective kits from Biozym Scientific GmbH (Hessisch Oldendorf, Germany) and submitted for image analysis with ImageJ [28].

*Data evaluation and statistical analysis:* Statistical analysis was performed with Prism 8 (GraphPad, San Diego, CA, USA). *p* values of less than 0.05 were considered statistically significant. Differences between the groups were tested with ANOVA or respective nonparametric methods (Kruskal–Wallis test), followed by multiple comparison (Dunnett’s or Dunn’s tests). Significance levels were marked by asterisks, where * corresponds to *p* < 0.05, ** *p* < 0.01, *** *p* < 0.001 and **** *p* < 0.0001.

## 3. Results

### 3.1. Characterization of SARS-CoV-2 VLPs

**Figure 1:**
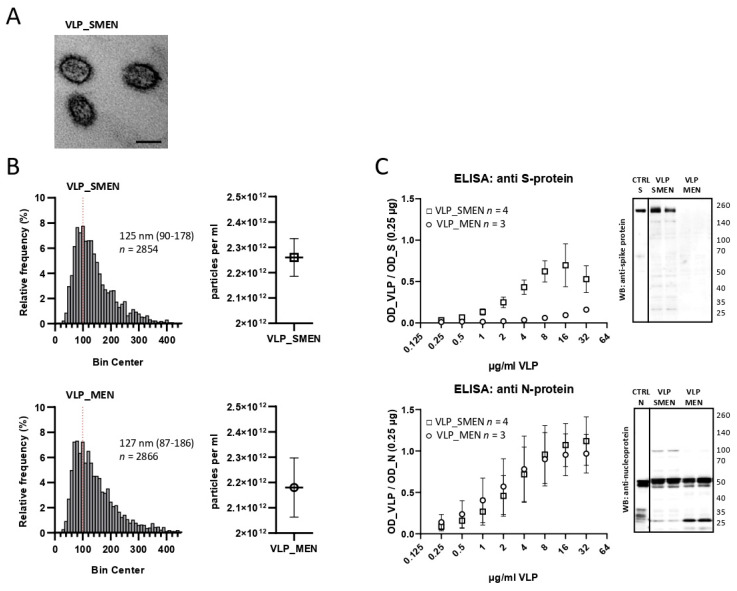
Characterization of VLP_SMEN and VLP_MEN. (**A**) Illustrative electron microscopy image of VLP_SMEN. Scale bar 100 nm (**B**) Nanoparticle Tracking Analysis (NTA). (**C**) ELISA using monoclonal antibodies against the spike or nucleoprotein and Western blot using a monoclonal antibody against the nucleoprotein and polyclonal human serum of a vaccinated individual.

The suspension-adapted Expi_MEN and Expi_SMEN cell lines were grown in 200 mL medium to a concentration of 3 × 10^6^ cells/mL. The production of smooth or spike protein containing SARS-CoV-2 VLPs was stimulated with 1 µg/mL doxycycline. The production of VLPs was paralleled by the decline of cell viability. At a viability of 40–60% the cell culture supernatant was cleared by centrifugation, filtered (0.45 µm), and subjected to tangential-flow filtration with a 300 kDa cut-off. Subsequently, the VLPs were precipitated by PEG and resuspended in sterile PBS. The successful assembly of particles with virus-like appearance was illustrated by electron microscopy (Figure 1A). Using nanoparticle tracking analysis (NTA), we determined a particle diameter of 125 nm 95% CI [90–178, *n* = 2854 particles] for VLP_SMEN and for the smooth VLP_MEN of 127 nm 95% CI [87–186, *n* = 2866 particles] (Figure 1B). VLP_MEN and VLP_SMEN were coated to the solid phase of a microtiter plate in a 2-log dilution series starting at 32 µg/mL. A monoclonal antibody dose-dependently detected the N-protein in VLP_SMEN and in VLP_MEN in an ELISA (Figure 1C). This finding was corroborated by the detection of the N-protein at the expected molecular weight in Western blot. As expected, the S-protein was only dose-dependently detected in VLP_SMEN and was absent in the smooth VLP_MEN in ELISA and Western blot.

### 3.2. Application of SARS-CoV-2 VLPs as Antigens for Serum Diagnostics

**Figure 2:**
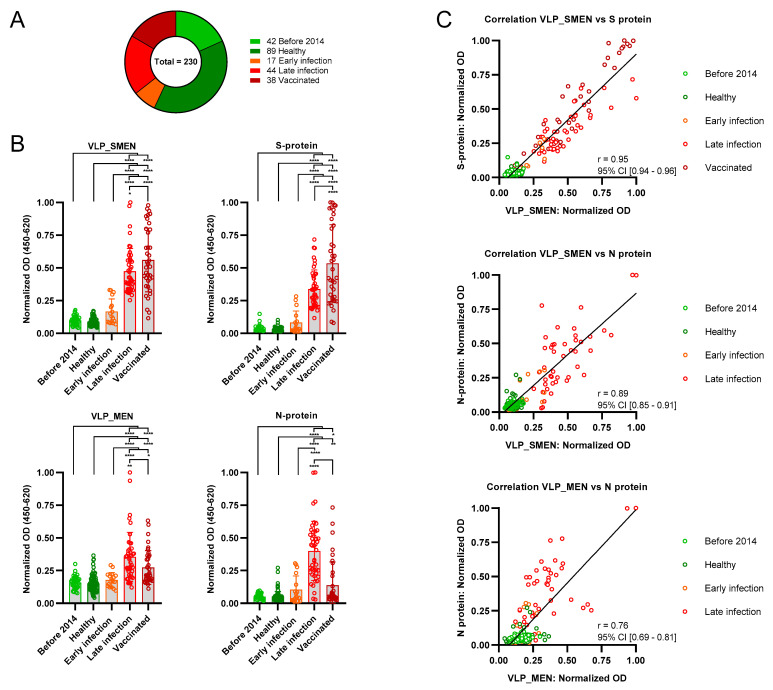
Serological characterization of 230 samples. (**A**) Classification of samples. (**B**) An in-house ELISA was used to assess the reactivity of the serum with VLP_SMEN, VLP_MEN, S- and N- protein. (**C**) Correlation between normalized OD values of VLPs and S- and N-protein. * *p* < 0.05, ** *p* < 0.01 and **** *p* < 0.0001.

In order to validate the performance of VLPs as antigens to detect SARS-CoV-2 specific immune reactions, we collected 230 serum samples (Figure 2A). This included 17 serum samples from patients at an early (<3 weeks) and 44 serum samples at a later time-point (>3 weeks) after SARS-CoV-2 infection. The serum collection took place between May 2020 and December 2021. Infection was verified by PCR or patient reports/documents of a positive PCR result that was obtained elsewhere. We also collected serum from 38 previously vaccinated individuals between March 2021 and December 2021. This group was not homogenous because some of the vaccinated patients reported a previous infection with SARS-CoV-2. As a negative control, 89 serum samples from healthy individuals without suspected SARS-CoV-2 infection were collected in May 2020, when an infection was also unlikely due to the early stage of the pandemic. Furthermore, we obtained samples from a cohort of 42 pregnant women that were collected before 2014. All 230 serum samples were analyzed with an in-house anti-human IgG ELISA using 1 µg/mL of either spike- or nucleoprotein or 5 µg/mL VLPs as antigen immobilized to a microtiter plate (Figure 2B). The VLP_SMEN coating solution contained approximately 0.12 µg/mL S-protein and 0.24 µg/mL N-protein as determined by quantitative ELISA against a 2-log dilution of the respective reference protein. The background signal of samples from healthy individuals and the pre-pandemic samples was generally low with all four antigens. One-way ANOVA revealed a significant difference between the serum groups for all four antigens. Tukey’s test for multiple comparison confirmed that the late stage serum samples exhibited significantly higher anti S-protein (*p* = 0.0001, 95% C.I. = −0.3646 to −0.1471) and anti N-protein (*p* < 0.0001, 95% C.I. = −0.3969 to −0.1954) IgG-levels than early-stage serum samples. Interestingly, we could also confirm that the anti-S-protein IgG levels are higher in vaccinated individuals than in those with a previous infection (Tukey’s test: *p* < 0.0001, 95% C.I. = −0.2815 to −0.1123). Within the vaccinated population, we found no difference in the anti-S-protein and in anti-VLP IgG levels between vaccinated individuals with or without a previous SARS-CoV-2 infection. A previous infection was inferred from a questionnaire and by the presence of anti-N IgG. All vaccines included in this study target only the SARS-CoV-2 S-protein. Using two times the mean OD of healthy individuals plus one standard deviation as a cut-off, it was possible to distinguish previously infected individuals from those that had only received the vaccination. This suggests that the use of the N-protein will receive more attention to distinguish SARS-CoV-2 infections from vaccine-elicited antibodies.

Pearson correlation coefficients were computed to assess the linear relationship between the normalized OD values of VLP_SMEN and the S-protein of all serum samples (Figure 2C). There was a positive correlation between the two variables, r(228) = 0.95, *p* < 0.0001. For the calculations of Pearson correlation coefficients comparing the normalized OD value of the N-protein to VLP_SMEN or VLP_MEN, the serum samples of vaccinated individuals were excluded. There was a positive correlation between the normalized OD value of the N-protein with VLP_SMEN, r(190) = 0.89, *p* < 0.0001 and VLP_MEN, r(190) = 0.76, *p* < 0.0001.

### 3.3. Comparison of the Performance of SARS-CoV-2 VLP-ELISA with a Certified IVD

**Figure 3:**
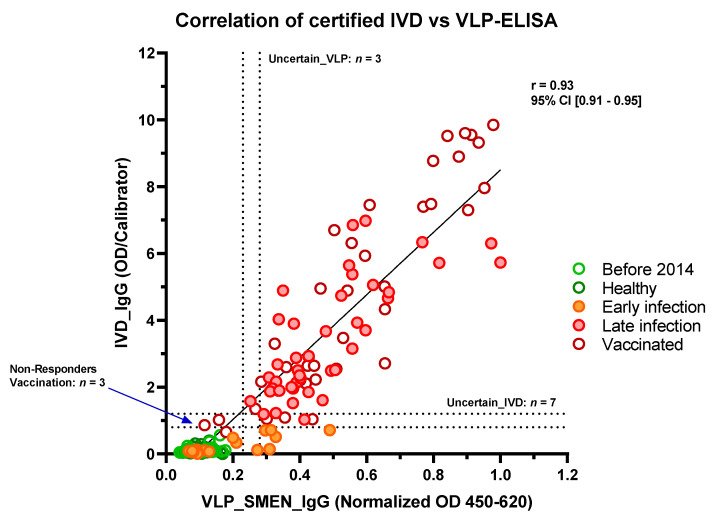
Comparison of the performance of SARS-CoV-2 VLP-ELISA with a certified IVD. Correlation of results from VLP-ELISA with a certified IVD.

Finally, we compared the VLP-ELISA with the performance of the N-protein or the full-length S-protein alone and a CE-certified IVD (EUROIMMUN, EI 2606-9601 G, Lübeck, Germany) (Figure 3 and Table 1). The IVD is an ELISA using the S1 domain of the SARS-CoV-2 S-protein as an antigen. In the VLP-, N- and S-ELISA, the cut-off that defines a “positive” sample was set as above two times the mean OD of healthy individuals plus one standard deviation (2OD+SD). “Negative” was set as below two times the mean OD of healthy individuals (2OD). Uncertainty (UC) was defined between the two cut-offs. The IVD and the VLP-ELISA correctly classified all pre-pandemic and healthy samples as negative, which accounted for a specificity of 100% (Figure 3 and Table 1). There were four and three false positive results for the S- and N-ELISA, respectively. The specificity was 96.1% for the S-ELISA and 95.4% for the N-ELISA. The diagnostic sensitivity for the 44 samples that were taken more than three weeks after the SARS-CoV-2 infection was 100% for the S-ELISA, 97.5% for the VLP-ELISA, 95.5% for the certified IVD, and 90.9% for the N-ELISA. Strikingly, the certified IVD did not identify any of the samples that were taken less than three weeks after the SARS-CoV-2 infection, whereas the N-ELISA identified 29.4% and the VLP-ELISA and the S-ELISA 35.3%. In total, there were seven cases of an uncertain result for the certified IVD, whereas the results from the VLP-ELISA were uncertain in only three cases. The combination of the low false positive rate and the high sensitivity suggest that there could be a benefit in using VLPs as antigens to identify SARS-CoV-2 specific immune reactions over individual antigens. 

## 4. Discussion

This is the first report using virus-like particles in a solid-phase immunoassay to detect antibodies against SARS-CoV-2. In contrast, VLPs are used in some vaccination approaches [18,29]. We were able to generate stable particles (127 nm for MEN VLP, 125 nm for SMEN VLP) of an expected size similar to the natural virus (60 to 140 nm as reported by [2]). The appropriate virus-like assembly was illustrated by electron microscopy. ELISA and Western blot experiments validated the presence of the N-and S-protein. The presence of the M- and E-protein can be implied because they are essential for the formation of particles [7,8,30,31].

Theoretically, there are some advantages to using VLPs instead of isolated single proteins or natural virus isolates to produce an antigen-coated surface for solid-phase immunoassays to detect anti-virus antibodies: (i) There is a greater likelihood that the antigens will retain their natural conformation. (ii) By utilizing the third dimension, there could be a greater amount of overall antigens available. (iii) Due to the lack of genetic material, VLPs are not infectious. (iv) When VLPs contain all of the virus proteins, some additional epitopes might be available. Therefore, an ELISA using VLPs may be able to detect more antibodies in a serum sample, resulting in a higher sensitivity. 

A similar VLP-based diagnostic ELISA was developed for the detection of human papillomavirus type 16, which is a risk factor for cervical cancer [10,12,32,33]. VLP-based ELISAs are frequently used in veterinary medicine to detect antibodies against different viruses such as swine vesicular disease virus [11], porcine circovirus type 2 [13], or Senecavirus A [14]. We were surprised that there is no VLP-based ELISA for the detection of antibodies against SARS-CoV-2 available today.

The VLP-ELISA that was developed in this study performed notably well. The small clinical study clearly demonstrated that the diagnostic performance met our expectations: 100% correct results for negative samples but higher sensitivity for positive samples compared to a commercially available CE-marked S-protein S1-subunit IVD kit (late infection 97.7% versus 95.5%, vaccinated 98.5% versus 84.2%). The sensitivity in the early stage (less than 3 weeks) of infection was remarkable: 35.3% versus 0.0%. It is important to minimize the diagnostic gap between the time of infection and the time of a positive antibody test. When considering the sensitivity and the specificity, the overall performance was better when using VLPs as an antigen rather than the individual N- or full-length S-protein alone.

Here we present a proof-of-concept for a VLP-based ELISA in SARS-CoV-2 infection. The preliminary clinical data show very high sensitivity and specificity. These data should be confirmed by a larger clinical study.

## Figures and Tables

**Table 1:** Comparison of the performance of the VLP-ELISA and a certified IVD.

	Before 2014	Healthy	Early Infection	Late Infection	Vaccinated
**No. samples**	**42**	**89**	**17**	**44**	**38**
^1^ E.I. pos	0	0	0	42	32
^1^ E.I. neg	42	89	17	0	1
^1^ E.I. UC	0	0	0	2	5
**% correct**	**100.0**	**100.0**	**0.0**	**95.5**	**84.2**
VLP pos	0	0	6	44	35
VLP neg	42	89	9	0	3
VLP UC	0	0	2	1	0
**% correct**	**100.0**	**100.0**	**35.3**	**97.7**	**92.1**
S pos	2	2	6	44	38
S neg	39	87	10	0	0
S UC	1	0	1	0	0
**% correct**	**92.9**	**97.8**	**35.3**	**100.0**	**100.0**
N pos	0	3	5	40	9
N neg	42	83	12	3	28
N UC	0	3	0	1	1
**% correct**	**100.0**	**93.3**	**29.4**	**90.9**	**23.7**

^1^ EUROIMMUN, EI 2606-9601.

## Data Availability

All related data and methods are presented in this paper. Additional inquiries should be addressed to the corresponding author.
